# What next for eating disorder genetics? Replacing myths with facts to sharpen our understanding

**DOI:** 10.1038/s41380-022-01601-y

**Published:** 2022-05-20

**Authors:** Laura M. Huckins, Rebecca Signer, Jessica Johnson, Ya-Ke Wu, Karen S. Mitchell, Cynthia M. Bulik

**Affiliations:** 1 Pamela Sklar Division of Psychiatric Genomics, Icahn School of Medicine at Mount Sinai, New York, NY 10029 USA; 2 Department of Psychiatry, Icahn School of Medicine at Mount Sinai, New York, NY 10029 USA; 3 Department of Genetics and Genomic Sciences, Icahn School of Medicine at Mount Sinai, New York, NY 10029 USA; 4 Icahn Institute for Genomics and Multiscale Biology, Icahn School of Medicine at Mount Sinai, New York, NY 10029 USA; 5 Seaver Autism Center for Research and Treatment, Icahn School of Medicine at Mount Sinai, New York, NY 10029 USA; 6 Mental Illness Research, Education and Clinical Centers, James J. Peters Department of Veterans Affairs Medical Center, Bronx, NY 14068 USA; 7 School of Nursing, University of North Carolina at Chapel Hill, Chapel Hill, NC USA; 8 Department of Psychiatry, University of North Carolina at Chapel Hill, Chapel Hill, NC USA; 9 National Center for PTSD at VA Boston Healthcare System, Boston, MA USA; 10 Department of Psychiatry, Boston University School of Medicine, Boston, MA USA; 11 Department of Medical Epidemiology and Biostatistics, Karolinska Institutet, Stockholm, Sweden; 12 Department of Nutrition, University of North Carolina at Chapel Hill, Chapel Hill, NC USA

**Keywords:** Diseases, Psychiatric disorders

## Abstract

Substantial progress has been made in the understanding of anorexia nervosa (AN) and eating disorder (ED) genetics through the efforts of large-scale collaborative consortia, yielding the first genome-wide significant loci, AN-associated genes, and insights into metabo-psychiatric underpinnings of the disorders. However, the translatability, generalizability, and reach of these insights are hampered by an overly narrow focus in our research. In particular, stereotypes, myths, assumptions and misconceptions have resulted in incomplete or incorrect understandings of ED presentations and trajectories, and exclusion of certain patient groups from our studies. In this review, we aim to counteract these historical imbalances. Taking as our starting point the Academy for Eating Disorders (AED) Truth #5 “Eating disorders affect people of all genders, ages, races, ethnicities, body shapes and weights, sexual orientations, and socioeconomic statuses”, we discuss what we do and do not know about the genetic underpinnings of EDs among people in each of these groups, and suggest strategies to design more inclusive studies. In the second half of our review, we outline broad strategic goals whereby ED researchers can expand the diversity, insights, and clinical translatability of their studies.

## Background

Over the past decade, substantial progress has been made in understanding the genetic etiology of anorexia nervosa (AN) and related eating disorders (EDs). Global collaborative efforts have yielded large-scale linkage, and later, genome-wide associations studies [1–4], revealing the first genome-wide significant loci [1] and 53 genes associated with AN across tissues [5]. These studies have led to new insights and hypotheses about the etiopathology of AN including evidence of psychiatric and metabolic risk factors [1]; shared genetic etiology with metabolic, anthropometric, and psychiatric traits [1]; and evidence for clinical consequences of predicted aberrant AN-gene expression [5]. Similar analyses of other EDs are underway. However, substantial unanswered questions remain about the biological mechanisms underlying AN and EDs; this gap in our understanding is a key contributor to the lack of effective, personalized treatments. ED science faces an uphill battle in replacing decades of myths, misunderstandings, and stereotypes about the presentation, demographics, symptoms, and etiopathology of EDs. Moreover, these misconceptions are subtly embedded in the research questions that have contributed to fundamental knowledge in the field and have influenced the populations we recruit and study.

To contextualize ED genetic research, we take as a starting point an aspirational document prepared and disseminated (available in 34 languages) by the Academy for Eating Disorders (AED), titled the Nine Truths About Eating Disorders [6]. Ongoing ED research is addressing several of the AED truths [7]. We focus on AED Truth #5 “Eating disorders affect people of all genders, ages, races, ethnicities, body shapes and weights, sexual orientations, and socioeconomic statuses” to illustrate how inclusivity in genetic research can contribute to a more comprehensive understanding of the full spectrum of EDs and ultimately revolutionize our understanding of this disease cluster.

AED Truth #5 directly counters many of the stereotypes that have hindered ED science. Dissecting this truth illustrates the importance of broad representation to prevent biased or distorted understanding of the causes and impact of EDs. The failure to acknowledge the diversity of individuals affected by EDs has led to narrow definitions of disease and diagnostic criteria, and consequently incomplete assessments of disease characteristics, risk factors, and etiology. Accordingly, individuals who fall outside of these parameters (i.e., those who are not young, thin, affluent white women) have been excluded from research and even from treatment. Stereotypes propagated by the media as well as the medical profession have contributed to under-detection and under-treatment of individuals with EDs who do not conform to these expectations. Under-detection, under-diagnosis, and under-referral for specialist treatment have been documented in men [8, 9], in people of color [10–14], in older individuals [15], in the LGBTQIA+ community [16–18], and in individuals living in larger bodies [19]. Failure to include these communities truncates our observations of risk factors and symptoms of EDs to a narrow slice of the affected population. Without representation, we cannot begin to understand the full spectrum of risk, and the mechanisms underlying EDs.

Expansion of genetic studies to include diverse presentations of EDs, and to capture the full spectrum of symptoms, behaviors, and outcomes that characterize EDs will yield increased power for discovery overall, but, more importantly, may also yield insights into specific genetic underpinnings of different facets of the disorders. Analytical approaches that can identify symptom-specific associations and account for potential biases can help reshape the understanding of EDs in the minds of researchers, clinicians, and the general public. In the Section “Background”, we outline what is known about EDs across the various groups referenced in AED Truth #5, and highlight existing genetic studies in these groups. In the Section “Large-scale approaches to address diversity in ED genetics”, we propose strategies for enhancing genetic research to advance the science of EDs.

### EDs affect people of all genders and sexual orientations

**Fig. 1:**
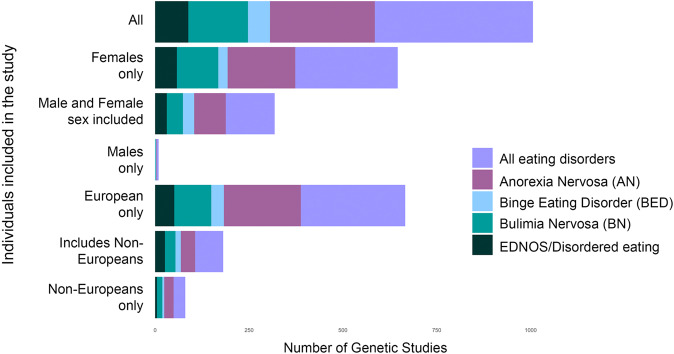
Historic imbalance in populations included in studies of eating disorders. Data include all genetic studies from 1980–2021. Studies where sex or race/ethnicity were not recorded are included in ‘all’. Studies with at least one male participant are included in ‘male and female sexes included’; studies with at least one non-European participant are included in ‘includes non-Europeans’. We caution that, for many of these studies, numbers of males/non-Europeans are very small.

ED research, diagnoses, treatment guidelines, and public attention have overwhelmingly focused on women and girls. Although men might be included in studies, numbers are often too small to warrant analysis or to generate confident conclusions (Fig. 1). Although EDs occur within and across gender and sexual orientation spectra [8, 16–18, 20], the underlying genetic and environmental risk factors may be very different within and across groups, necessitating specialized screening, prevention, and treatment. In this review, we use ‘sex’ to refer to categorical variables including ‘male’ and ‘female’ as defined in the original studies, and ‘gender’ to refer to socially constructed behaviors and identities, rather than biological attributes [21, 22].

#### EDs in boys and men

According to a recent large meta-analysis, the gender ratio for AN is estimated to be 7:1 [23], for bulimia nervosa (BN) ~3.2:1 [23], and for binge-eating disorder (BED) 2.8:1 [23]. The lifetime prevalence of EDs was 8.4% for women and 2.2% for men around the world, based on an analysis of 94 articles with ED diagnoses from 28 countries (e.g., Argentina, Brazil, Canada, China, Finland, Germany, Italy, Japan, and Portugal) [23]. Globally, the lifetime prevalence of AN was 1.4% for women and 0.2% for men; BN was 1.9% for women and 0.6% for men; and BED was 2.8% for women and 1.0% for men [23]. These figures reflect diagnostic criteria developed based on clinical presentation in women and likely underestimate the prevalence in men. For example, whereas women (in Western countries) address their underlying “drive for thinness” and concern with weight and shape by focusing on being thin and losing weight, men tend to show more concern for building lean muscle [24] and express that drive via reducing body fat percentage or striving for muscle definition. Compensatory behaviors also differ, with women being more likely to engage in self-induced vomiting and diuretic/laxative use, whereas men are more likely to exercise excessively and take anabolic steroids or supplements to pursue their ideal body shape [25]. In addition, the long history of EDs being considered a female disorder increases hesitancy and shame in males decreasing help-seeking [24], and hinders detection by clinicians who do not include EDs on the differential for male patients [9].

#### EDs in the LGBTQIA+ population

LGBTQIA+ individuals report increased prevalence of psychiatric disorders, including all EDs [16–18], due at least in part to the systematic discrimination, harassment, and violence they experience [16–18, 26]. Watson, et al. [17], found that “enacted stigma” (the collective experience of systematic discrimination) was associated with multiple disordered eating phenotypes such as binge eating, fasting, and vomiting. Subsequent studies have confirmed internalized trans- and homophobia as risk factors for EDs [26]. Underlying facets of body image and shape concerns as risk factors for EDs may differ dramatically between transgender and cisgender individuals [26, 27]. Alignment with specific gender presentations may present as a key motivation for disordered eating [26–29]; for example, the desire to suppress menstruation through restriction and excessive exercise has been observed in transgender men [27, 30].

#### What we know and do not know about ED genetics relative to gender and sexual orientation

Genetic studies have identified both shared and distinct genetic risk factors across sexes. For example, female relatives of men with AN have an elevated crude relative risk of AN (20.3) [31], implying at least some shared genetic risk. However, studies comparing same- and opposite-sex twins found that only 50% of the genetic risk for ED behaviors was shared between the sexes, with females having a higher loading of genetic risk [32]. Heritability patterns also differ developmentally: twin studies of boys and young men estimate ~50% heritability across pre-pubescence, adolescence, and into adulthood; whereas, heritability among girls and young women was undetectable (~0%) until puberty, reaching 50% only in adulthood [33]. More recent studies have leveraged genetic correlation analyses to examine sex-specific relationships between AN and anthropometric traits. For example, AN and body fat percentage (BF%) are more highly genetically correlated among females than among males [34, 35], and partitioned heritability analyses of *SNP-h*^2^ showed that BF%_female_ was significantly enriched for CNS tissue while BF%_male_ was enriched for adipose tissue [35].

No explicit research exists on the genetic risk of EDs among LGBTQIA+ individuals, though we can reasonably assume that many of the same genetic influences on EDs are acting on these individuals as cisgender and heterosexual individuals, although environmental risk factors may differ. What is essential for ED genetic studies is that these individuals are included and unique risk factors that contribute to their experience of EDs are considered and assessed.

It is overly simplistic to conclude that AN and anthropometric traits have different genetic risk factors between men and women. Such an interpretation ignores the historical bias in ED research. Because diagnostic guidelines were developed and tailored to the presentation of EDs in women and girls, assessment instruments likely have differential accuracy and sensitivity according to sex and gender [36]. Moreover, heritability and expression of a trait do not occur in a vacuum. Diagnosis of a disorder (and consequent inclusion in research) is representative not just of genetics and downstream biology, but of a constellation of social and environmental exposures, including ED risk factors, but also vigilance of caregivers and clinicians to ED symptoms, and access to appropriate treatment. Similarly, studies of anthropometric traits will likely be confounded by gendered societal pressure for thinness, such that women are exposed to higher rates of bullying, shame, and pressure to lose weight. These factors might account for the brain-specific heritability of body fat percentage in women [34]. Such considerations are key to introducing equity into studies of EDs, and will be notable throughout our discussions not just of gender, but of race and ethnicity, age of onset, and socio-economic status. Finding ways to address, account for, or remove these factors will be key to furthering our understanding of EDs.

### EDs affect people of all races and ethnicities

#### ED prevalence across races and ethnicities

The limited available data from population-based studies report few racial or ethnic differences in the prevalence of EDs among adults, although results are inconsistent. Nationally representative studies in the US report no significant racial or ethnic differences in the prevalence of AN, BN, or BED [8], although more recent studies report significantly lower lifetime prevalence of AN among Black and Hispanic/Latinx participants [37], higher prevalence of BN among Hispanic/Latinx adolescents [38], and higher prevalence of general ED pathology among American Indian/Alaskan Native and Hispanic/Latinx college students [39]. There have been few representative, population-based studies of ED prevalence outside of the U.S., Europe, and Australasia. The World Health Organization World Mental Health surveys reported prevalence estimates for BN and BED from 14 countries, including Columbia (0.4% BN, 0.9%, BED), Brazil (2.0% BN, 4.7% BED), Mexico (0.8% BN, 1.6% BED), Romania (0.0% BN, 0.2% BED), Belgium (1.0% BN, 1.2% BED), France (0.7% BN, 1.7% BED), Germany (0.3% BN, 0.5% BED), Italy (0.1% BN, 0.7% BED), the Netherlands (0.9% BN, 0.9% BED), New Zealand (1.3% BN, 1.9% BED), Northern Ireland (0.5% BN, 1.5% BED), Portugal (0.8% BN, 2.4% BED), Spain (0.7% BN, 0.8% BED), and the U.S. (1.0% BN, 2.6% BED) [40]. A 2013 review of prevalence investigations of EDs included studies from the World Mental Health surveys, the U.S., Western Europe, Latin America, South Korea, and New Zealand. The authors reported that the pooled prevalence of lifetime EDs was higher among Western countries (1.29%) compared to the South Korean sample (0.21%) [41]. A review of epidemiological studies of EDs in African countries found that most included relatively small community or student samples. No cases of AN were reported. The pooled prevalence of BN was 0.87%, and the pooled prevalence of Eating Disorders Not Otherwise Specified was 4.45% [42].

Furthermore, studies have reported differing prevalence of component symptoms of binge eating, purging, body dissatisfaction, and fear of weight gain across races and ethnicities. For example, Asian men and women with EDs tend to exhibit lower fear of fatness [43–45], a key DSM-5 diagnostic criterion, as well as higher levels of thin ideal internalization compared to European Americans and African Americans [46], possibly influencing ED prevalence estimates among Asian men and women.

#### Detection and referral and assessment of EDs in diverse populations

Differences in prevalence estimates may reflect that people of color are less likely to be diagnosed with an ED, to seek treatment, and to be referred for specialist treatment [10, 13, 14]. As outlined in the section on gender and sexual orientation, historical biases in ED research have yielded diagnostic schema and treatment guidelines based on very specific presentations. Diagnoses and guidelines may disproportionately address clinical features expected in a prototype white cisgender female patient, reducing applicability across racial and ethnic groups. Similarly, cultural differences in environmental exposures and racial/ethnic differences in ED symptomatology are rarely captured by existing ED assessments. Studies using established assessments are able to say whether individuals report standard symptoms, but inadequately address unique symptoms or risk factors that may characterize the illnesses across diverse groups—again potentially hampering knowledge and perpetuating health disparities.

#### What we know and do not know about ED genetics relative to race and ethnicity

It is widely known that complex trait genetics has focused overwhelmingly on individuals of European ancestry [47, 48], including over 90% of individuals in psychiatric genetics GWAS [49]. Large GWAS of non-Europeans (e.g., the PAGE consortium [50]) have enabled trans-ancestry fine-mapping and led to discovery of novel disease-associated genes, even using substantially smaller sample sizes [50, 51]. Although relatively underpowered, multi-ancestry sequencing studies of schizophrenia indicate that rare variant burdens are similar across ancestries [52]. The success of cross-ancestry GWAS and sequencing studies in these traits shows the promise of trans-ancestry fine-mapping loci using diverse cohorts and may increase the likelihood that causal variants can be found [49–51]. Although early work on candidate genes, genome-wide microsatellite studies, and replications emerged from Japan [2, 53, 54], subsequent AN GWAS have been restricted to European-ancestry populations. Although large diverse samples are not yet available in EDs, this is poised to change as the ED Working Group of the Psychiatric Genomics Consortium (PGC-ED) has highlighted diversification of samples and engagement of international researchers as a priority [55].

### EDs affect individuals across the lifespan

EDs affect people of all ages [15], although symptoms, presentation and prevalence may change over the lifespan. As with gender and race, a historical focus on a specific presentation (i.e., young girls and women) may have introduced age bias into diagnostic criteria, assessment instruments, and treatment guidelines. Across the lifespan, disparate genetic and environmental risk factors may underlie superficially similar ED behaviors. Investigating genetic factors underlying motivations and emotions related to EDs, as well as specific behaviors and anthropometric phenotypes across the lifespan may provide novel insights into the psychiatric and metabolic causes and mechanisms underlying EDs.

#### EDs in youth

Disordered eating behaviors occur in young children, but can differ in clinical presentation. For example, “avoidant/restrictive food intake disorder (ARFID)” [56] occurs in 1.5–3.2% of children and adolescents [57, 58], and is characterized by food restriction for a variety of non-weight related reasons, including sensory sensitivities (e.g., texture aversions), fear of choking or vomiting (i.e., phobic avoidance), or low interest in food and low appetite [59]. The restriction associated with ARFID does not include fear of weight gain or body dysmorphia [59] as in AN. Similarly, loss-of-control eating (LOC), a clear precursor to binge eating [60] occurs in more than one quarter of children with overweight or obesity [61], and is characterized by feeling unable to control what or how much one is eating [62, 63]. Although both ARFID and LOC can occur alone, they also commonly co-occur with other psychiatric disorders. The risk of LOC is elevated in children with ADHD [64], anxiety [65, 66], and in children who have experienced weight-related bullying [67], and the risk of ARFID is elevated among children with autism spectrum disorder [68], and vice-versa [69]. Moreover, both LOC and ARFID exhibit complex relationships with metabolic factors: for example, youth with LOC who showed greater anxiety had higher leptin levels [65] and more frequent metabolic syndrome [66]. The extent to which these childhood presentations are precursors for later AN, BN, and BED is not entirely clear; however, ongoing developmental research aims to characterize developmental continuities and discontinuities in EDs.

#### EDs in midlife and beyond

EDs that occur in midlife and beyond can represent persistent cases, relapsing cases in individuals with initial onset in childhood or adolescence, or most uncommonly, new onset cases [70]. The requirement for an extremely low BMI in AN diagnosis likely biases toward a younger cohort, as BMI naturally increases with age [71, 72], perhaps explaining patterns of earlier AN incidence (peaking at 16 [73]) compared to Other Specific Feeding and Eating Disorder (OSFED; peak 18–30 [73]). Further, inclusion of amenorrhea in diagnostic criteria naturally biases clinicians towards people who menstruate i.e., missing pre-pubertal and post-menopausal females and males. EDs do, however, occur in midlife and beyond. In fact, Eating Disorder Examination-Questionnaire (EDE-Q) scores among women do not decline until after age 54, whereas scores in men peak at ages 55–64 [74]. Further, 15.7% of respondents aged 40–60 have clinically meaningful EDE-Q scores [75], and 13% of women aged 50+ report at least one core ED symptom [76]. Moreover, familiar factors are cited as contributors, including self-esteem [75, 77], body dissatisfaction [78, 79], BMI [79, 80], perfectionism [78], and societal pressure for thinness [78].

#### What we know and do not know about ED genetics across the lifespan

Little is known about the genetic risk factors underlying ED onset at different ages. A recent GWAS examined genetic etiology of AN age-of-onset, and compared early onset (defined as <13 years of age) to typical-onset AN [81]. Distinct genetic risk factors were associated with early onset compared to typical onset, including a potentially causal correlation between younger age at menarche and early onset, and associations between typical age of onset and a range of anthropometric traits [81]. No explicit research exists on the genetics of age-of-onset in other EDs; nor have any studies to our knowledge investigated the genetics of later onset AN. However, given the shared and distinct features of EDs across the lifespan, including age-diverse samples will be essential to capture context- and developmentally-specific differences in environmental and genetic risk factors.

### EDs affect individuals across socioeconomic groups

EDs affect individuals at all socioeconomic status (SES) levels. However, individuals with lower SES are less likely to receive screening or treatment for EDs and thus remain underrepresented among clinical samples [82], particularly in the U.S. Although early findings suggested that AN was associated with higher SES [83], a more recent systematic review of U.S. and Europe community and population-based studies concluded that EDs are not disorders of affluence [84]. Further, in a cohort of two million men and women in Sweden, a country with free or low-cost healthcare, an ED diagnosis was associated with parent education but not income after adjusting for education, suggesting that SES was not systematically associated with seeking healthcare for EDs [85]. Little is known regarding associations between EDs and SES in non-Western countries, likely due to the paucity of prevalence studies as discussed earlier. For example, in the 2010 Global Burden of Disease study, regions other than North American had poor coverage of epidemiological data on EDs [86]. In the aforementioned World Mental Health surveys, only one country (Columbia) was considered low-income, while the other 13 countries were upper-middle to high-income [40]. However, this study did not statistically compare prevalence across countries. In a study of National Health Insurance data from Taiwan, which includes 99% of the Taiwanese population, findings indicated that the incidence and prevalence of EDs had increased over the previous decade. However, estimates were lower than those obtained from community samples, suggesting that factors other than treatment affordability may have impacted treatment-seeking in this sample [87].

Other factors related to lower SES may be associated with increased risk for EDs. Some studies in U.S. samples have found evidence that food insecurity or insufficiency is associated with BED and BN [88, 89], while stress and childhood adversity are associated with ED symptoms, further underscoring the need to ensure that individuals across the SES spectrum are represented in ED studies and treatment settings [90, 91].

#### What we know and do not know about ED genetics and SES

Indicators of SES, including education, social deprivation, and household income, have heritable components [92], and studies have revealed genetic correlations between SES and psychiatric disorders [93]. A recent study found that SES was positively genetically correlated with AN [94]. Partitioning out the genetic SES variance resulted in reduced genetic variation for AN, and reduced genetic cross-trait associations among psychiatric disorders [94]. These results emphasize the importance of controlling for SES in order to reduce bias in estimates of genetic variance. A twin study revealed that neighborhood disadvantage was associated with increased disordered eating in girls across all stages of pubertal development, and the expected pubertal increases in genetic influences on disordered eating were only observed in girls from advantaged backgrounds [95]. Genetic influences on disordered eating were potentiated much earlier for girls living in disadvantaged contexts, suggesting interplay between genetic risk and SES.

### EDs affect individuals of all body shapes and weights

Disproportionate attention to AN has obscured the fact that EDs occur in individuals of all body shapes and sizes. Although low BMI is required for a diagnosis of AN, BN can occur in individuals across the BMI spectrum, and BED occurs in individuals with typical and higher weight bodies. The DSM-5 now recognizes atypical AN (AAN) in which individuals meet all diagnostic criteria for AN including weight loss but do not present with low weight [20, 96]. AAN has been associated with poor nutritional and medical status secondary to weight loss [97], poor quality of life [98], premorbid overweight and obesity [99], and history of weight-based teasing [100]. Despite this, individuals with AAN are less likely than those with AN to be screened for and to receive treatment [101, 102]. Considerably more work is required to understand the full array of presentations of AAN across the diverse populations highlighted in this review.

#### What we know and do not know about ED genetics relative to body shape and weight

Genetic research has addressed body shape and weight in three ways. First, GWAS have highlighted shared genetic factors between AN and body shape and weight, including negative genetic correlations between AN and body fat percentage (*r* = −0.36), fat mass (*r* = −0.33), BMI (*r* = −0.32), waist to hip ratio (r = −0.2), hip circumference (*r* = −0.2) and waist circumference (*r* = −0.24) [1], suggesting that many of the same genes that increase risk for AN also contribute to these anthropometric traits. However, existing GWAS have focused exclusively on AN in individuals with low BMIs, leaving individuals with EDs in larger bodies unstudied (although such studies are underway). Second, applying polygenic risk scores (PRS) in biobank data demonstrates that AN-PRS are associated with weight loss, even among adults who have never been diagnosed with AN [103]. Further, while AN, BN, and BED have similar psychiatric trait associations, they diverge in their associations with metabolic and anthropometric traits [104]. Although AN and anthropometric measures are negatively correlated, BED is significantly positively associated with several body shape and weight measures, suggesting that BMI-associated genomic variants are broadly relevant for all three EDs, but may act in opposite directions [104]. Third, phenome-wide association studies (PheWAS) showed that AN-genes are associated with anthropometric traits (including lowest recorded weight and weight change over time) in a clinical cohort with no history of AN; further, AN-gene associations with chronic pain, substance use and cholesterol levels were mediated by BMI [5].

In addition, genetic research has the potential to shed light on the extent to which restrictive EDs in individuals with larger bodies (i.e., AAN) are genetically similar to AN. Comparing GWAS of AN and AAN could inform nosology and shed light on whether it is actually appropriate (from a genetic perspective) to label this presentation as a form of AN. Divergent results could inform whether AN and AAN share the same underlying genetics and only differ in BMI or represent genetically distinct syndromes.

### Large-scale approaches to address diversity in ED genetics

**Fig. 2:**
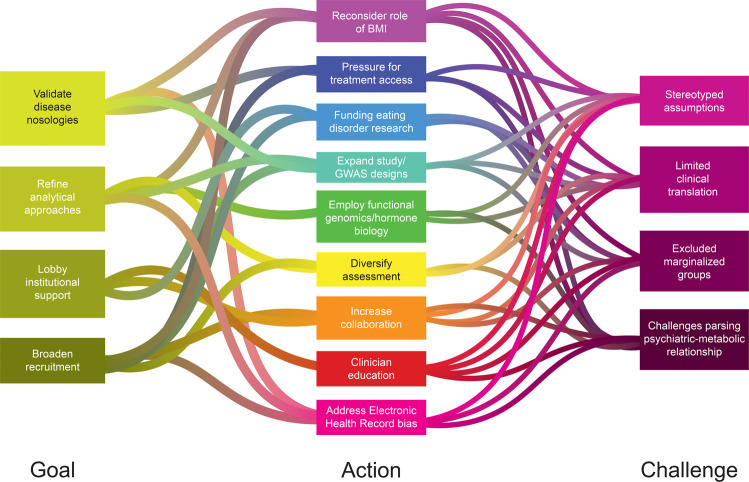
Proposed strategies to address key needs and existing challenges in eating disorder research. Here we diagram the broad goals and specific actions we propose to address and the current challenges in eating disorder research. More detailed descriptions of the challenges and recommended actions are addressed in this review.

In the Section “Background”, we outlined ways in which stereotypes and misconceptions have biased our understanding of and research on EDs. Here, we suggest five overarching foci to improve the applicability of genetic research on EDs to the full spectrum of afflicted individuals (Fig. 2).

### Broader and more inclusive recruitment

As there is no guarantee that the same genetic and environmental factors influence risk for EDs across demographic groups, innovative practices are essential to ensure representation of individuals of diverse ancestry, gender and sexual orientation, age, SES, and body sizes. Strategies to recruit and retain diverse participants have been discussed in detail elsewhere [105], but several core points warrant emphasis. First, participants report that seeing themselves represented in study personnel and in recruitment materials and websites increases interest in participation. Likewise, participants who share their personal stories about being from a minoritized group and suffering from an ED can help reduce shame and encourage participation. A clear explanation of why representation is important can also help potential participants decide to volunteer. Many fear that researchers desire for inclusivity is simply to tick a demographic checkbox, but feel more inclined to participate when they learn that their participation will contribute to ensuring that any treatments that emerge from the science will be relevant to others who share their background. Understanding the actual downstream implications of participation can be a powerful incentive to join a study. Finally, engaging participants in the process from the earliest phases of study design (i.e., co-design [106]) and providing regular updates about the study progress (websites, blogs) creates a sense of ownership and community that allows participants to feel like part of a larger team.

### Building capacity

The ED research workforce is overwhelmingly white, which is also true for the ED genetics workforce. Building capacity to diversify the workforce has to start early in the educational trajectory. Recruiting interested individuals into science, technology, engineering and mathematics (STEM) as early as middle school (~ages 11–14) and engaging them in research related to eating, body composition, nutrition, psychology, psychiatry, and EDs will ensure a more diverse workforce in the future. Moving away from a purely sociocultural theory of ED etiology will engage more individuals interested in science (including genetics) and encourage more research on the biology of EDs.

### Restructuring diagnostic guidelines and disease nosology

A recurring theme in Part 1 was the extent to which the narrow focus on young white thin females with AN in both research and in lay circles has shaped and biased the diagnostic schema, instruments, and research conclusions in the ED field. This restricted perspective has considerable downstream implications—from individuals outside of this stereotype who are neither detected nor referred for treatment, to who volunteers to participate in studies, to the conclusions that are drawn from samples that are mostly of European ancestry. The bias is compounding because any findings from these samples only serve to reinforce existing stereotypes about disease prevalence and presentation. Failure to include people of color and of size, other genders, and children and older people in our research studies severely limits our understanding. Moving the field forward will require a careful reconsideration of disease nosology and treatment guidelines. Guidelines should be restructured to consider differing presentations, behaviors, environmental risk factors, and psychological drivers, bearing in mind that many of the marginalized identities discussed in this review are intersectional, meaning that diagnostic criteria need to be flexible and applicable simultaneously to many different groups and presentations.

Researchers should also attend to nomenclature used outside of the DSM and ICD that often emerges from traditional and social media to describe dysfunctional eating behavior. Popular terms like disordered eating, body dysmorphia, food addiction, orthorexia, and relative energy deficiency in sport (previously female athlete triad) often capture trends in the population that describe what people are actually experiencing. The breadth in naming and lack of parallel to current treatment guidelines presents a variety of challenges. First, by using the above terms in the medical literature without defined criteria, we exacerbate challenges in clinical translation, as findings related to disordered eating might not easily be applied to a patient with AN or BN presenting to clinic. Second, we may unknowingly exclude historically marginalized groups who might find one of the above terms to be more representative of their lived experience. For example, if an individual identifies with a diagnosis of body dysmorphia, but researchers only recruit patients with atypical AN, we may miss including that participant and others like them. Lastly, the ED field suffers from chronic underfunding due in part to a dilution of recognition of the true prevalence of the disorders and resulting public health impact [107]. By addressing the many names and experiences lived with ED we can improve treatment access to the public and improve funding and discovery in research.

Success in this area will require a multi-pronged approach. First, the creation and deployment of more broadly useful screening tools and questionnaires should include opportunities to describe symptoms and behaviors falling outside stereotyped expectations for each condition; should not include BMI stratification or BMI-based inclusion/exclusion criteria; and should be appropriate cross-culturally.

Researchers should also explore alternative or additional phenotyping approaches that rely on observed behaviors, specific symptoms, and digital phenotyping. Other examples include electronic-health record-based phenotype inference [108–110] or analysis of specific reported symptoms and behaviors from population and biobank studies. However, we caution that these approaches are also susceptible to bias; creating phenotype algorithms without first interrogating any assumptions and implicit biases risks automating inequalities [11, 111]. For example, a previous study used 18 ICD codes to identify potential AN cases in a large healthcare system [71], selecting positive cases for further chart review. However, 6/18 codes related or referred to menstruation (despite the demonstrated ineffectiveness of amenorrhea as a diagnostic criterion [112–115]), substantially biasing the sample towards people who menstruate.

Researchers should leverage insights from genetic studies to reshape disease nosology. Novel analytical approaches such as pathway-based PRS, transcriptomic imputations, and incorporating electronic and longitudinal health records afford researchers an unprecedented opportunity to study disease on an individual rather than population level. Such analyses might allow researchers to infer molecular subtypes of disease, identifying for example clinically-relevant subgroups associated with specific molecular signatures. Alternatively, these analyses might reveal that clinically distinct presentations are not rooted in genetic differences, rather stemming from societal or environmental exposures. Any assessment of environmental risk factors or correlates must account for the broad range of experiences that individuals with EDs from diverse backgrounds might have. Beyond the historical focus on the societal thin ideal, space must be made for assessing main effects of group-specific or shared factors (e.g., weight-based stigmatization or discrimination, food insufficiency, teasing and bullying, race-based discrimination) as well as gene x environment interplay. Understanding these disparate presentations, behaviors and symptoms will also represent a significant step towards personalizing treatment options, effectively identifying interventions and therapies that match specific symptoms and behaviors.

### More sophisticated analytical approaches to refine phenotype-genotype associations

Although there are substantial insights to be gained from larger, more inclusive GWAS of AN, it is imperative that we expand our analytical approaches, adopting cutting edge statistical techniques in order to more fully elucidate the genetic architecture of EDs. Elegant and intentional study design may increase our power to understand disease as much as increasing sample size.

Substantial attention should be paid to approaches that can identify shared and distinct associations between ED diagnoses, or within disorders between subtypes. Explicit comparison of cases between psychiatric disorders has previously been shown to elucidate disorder-specific genic associations [116]. As such, genetic studies that compare and contrast AN and BN, for example, might increase our power to detect genetic correlates of shared and distinct symptoms and behaviors. These studies might include joint analysis across subtypes; case-case GWAS that explicitly compare individuals with different ED subtypes; PRS or LDScore approaches, or next generation analytical approaches such as genomic structural equation modeling (SEM). Further, these studies may also bring us closer to clinical utility: the diagnostic challenge for a clinician is rarely ‘whether’ an individual will develop a disorder: rather, the challenge is to identify the correct diagnosis among several similar possibilities, or to predict disease prior to onset. For this goal, differentiating AN from BN will be more useful than AN from controls.

Researchers should also investigate the role of potentially clinically relevant endophenotypes. For example, comparing genetic risk factors across the lifespan may elucidate relationships with specific biological pathways and mechanisms. Studies of younger cohorts may reveal the role of metabolic and hunger/satiety dysregulation in AN, whilst examining ED onset in older cohorts may elucidate specifically the role of psychiatric pathways. Investigating these groups may also yield insights into the role of hormones associated with menarche and menopause to ED pathology through BMI and/or body dissatisfaction [117]. A secondary analysis of a genome-wide association study of AN with 9,335 cases and 31,981 controls found that early-onset AN was significantly genetically correlated with younger age at menarche, and Mendelian randomization analysis supported a causal link between younger age at menarche and early-onset AN [81]. Further work needs to be completed to determine the hormonal relationship between estrogen fluctuations, appetite and satiety hormones, BMI, body satisfaction, and psychiatric phenotypes.

Researchers should also undertake approaches that can identify clinical consequences of genetic associations. For example, phenome-wide association studies (PheWAS) test for associations between genetic risk factors and the full patient phenome, including diagnostic records, reactions to medications, substance use/abuse, and longitudinal trajectories. These approaches might be particularly useful in disentangling whether specific comorbidities occur due to genetic pleiotropy (i.e., shared genetic underpinnings), healthcare pleiotropy (i.e., due to common mis-diagnosis, or as common ‘stops’ on a diagnostic trajectory), or due to diagnostically meaningful overlap.

### Expand insights beyond genotype, towards biology and environmental interactions

Approaches that rely on genotype alone will ultimately be limited in the potential for translational and clinical insights. A full dissection of potential functional genomics approaches and integrative analyses is outside the scope of this review, and indeed has been explored in detail elsewhere [118–122]. However, we caution that researchers must consider factors beyond genetics in their interpretations of GWAS and consequent associations. Failure to consider the biological, environmental, and societal contexts under which genetic studies are conceived, recruited, and analyzed risks mis-interpreting or over-interpreting the relevance of our findings, and potentially entrenching bias and stereotypes throughout our research. Put simply, expanding our samples and approaches will be pointless if we do not also consider carefully all the implications of our findings.

We should be wary of over-interpreting differential heritability, correlations, or relative lack of genome-wide associations between two groups. For example, it might be tempting to interpret differential heritability between sexes as explaining sex differences in ED diagnoses. However, genetic associations are only as good as the phenotypes we provide; researchers should consider that phenotyping instruments might have differential power; diagnostic contamination might occur at different rates; disease severity might be substantially different between groups; and study participation rates may differ dramatically. If indeed researchers identify true sex-specific effects (or other differential associations across groups) that are not confounded by diagnostic differences, it is important not to default to assumptions of genetic essentialism [22]. Instead, the next step must be to consider how these interact with biology and environment to cause disease; for example, considering the effects of specific variants on hormone biology, age at menarche, or related factors that might influence ED development. Understanding the role of these environmental contexts will be key in pinpointing appropriate treatments in differing life stages and contexts, and perhaps in understanding why some treatments are less effective in certain patient groups.

Consideration of the impact of societal factors is also essential. These may include exposure to stress and early life adversity; access to therapy and/or healthcare; access to healthy foods and opportunities for exercise; and support networks of family and friends. On the surface these factors are not genetic; however, it is likely that they correlate with genetic factors, as evidenced for example by recent GWAS of SES [94, 123] and loneliness [124]. Most obviously, healthcare access differs substantially among countries, and will likely influence disease trajectory, diagnostic rates, and study participation. Even within countries, systemic racism prevents equal access to early life support, healthcare services and referrals to specialists, and may increase exposure to a variety of disease risk factors [11, 12, 111, 125–132]. These factors may confound our studies, with the degree of confounding significantly correlated with genetic factors. As such, we might expect our GWAS and genetic studies to be differentially powered across ancestries, as increased environmental risks in one group might decrease our ability to detect genetic associations [11]. These issues should be borne in mind when interpreting GWAS associations and underscore the need to collect large and diverse GWAS samples.

Finally, we note that expanding inclusion within our studies is vital to equity in research and clinical care. The ability to participate in studies and research, and access to the findings and benefits of that research, should be equally available to all. Increasing diversity will inarguably increase our insight and understanding of ED biology, and indeed of psychiatric disease genetics more broadly; however, the goal of inclusion and equity alone is a sufficient motivator. Research conclusions that emerge from the study of a narrow and privileged subset of individuals with EDs and the treatment approaches they generate overestimate our actual understanding of disease and further perpetuate already damaging health disparities. This work is both hard and expensive, but must be a priority to replace myths with facts and to sharpen our understanding of EDs.
